# ﻿A new species of Asiatic shrew of the genus *Chodsigoa* (Soricidae, Eulipotyphla, Mammalia) from the Dabie Mountains, Anhui Province, eastern China

**DOI:** 10.3897/zookeys.1083.78233

**Published:** 2022-01-25

**Authors:** Zhongzheng Chen, Tingli Hu, Xiaoxin Pei, Guangdao Yang, Fan Yong, Zhen Xu, Weiying Qu, Kenneth O. Onditi, Baowei Zhang

**Affiliations:** 1 Collaborative Innovation Center of Recovery and Reconstruction of Degraded Ecosystem in Wanjiang Basin Co-founded by Anhui Province and Ministry of Education, School of Ecology and Environment, Anhui Normal University, Wuhu, Anhui 241002, China Anhui Normal University Wuhu China; 2 School of Life Sciences, Anhui University, Hefei, Anhui 230601, China Anhui University Hefei China; 3 Forestry Investigation and Planning Institute of Anhui Province, Hefei, 230001, Anhui, China Forestry Investigation and Planning Institute of Anhui Province Hefei China; 4 Research Center for Nature Conservation and Biodiversity, Nanjing Institute of Environmental Sciences, Ministry of Ecology and Environment, Nanjing, Jiangsu, 210042, China Ministry of Ecology and Environment Nanjing China; 5 Kunming Institute of Zoology, Chinese Academy of Sciences, Kunming, Yunnan 650204, China Kunming Institute of Zoology, Chinese Academy of Sciences Kunming China

**Keywords:** *Chodsigoadabieshanensis*, molecular analysis, morphology, new species, taxonomy

## Abstract

Asiatic shrews of the genus *Chodsigoa* (Soricidae, Eulipotyphla) currently comprise nine species, mostly occurring in southwest China. From May 2017 to August 2020, 11 specimens of *Chodsigoa* were collected from the Dabie Mountains in Anhui Province, eastern China. Their morphology was compared with other species within the genus and one mitochondrial (cytochrome b) and two nuclear (apolipoprotein B and breast cancer 1) genes were sequenced to estimate the phylogenetic relationships of these specimens. Based on morphological and molecular evidence, these specimens are recognized as a distinct species, *Chodsigoadabieshanensis***sp. nov.**, which is formally described here. Morphologically, the new species is most similar to *Chodsigoahypsibia*, but it is distinguishable from all known congeners by the combination of dark brownish pelage, small size, and relatively short tail. Phylogenetic analyses revealed that *C.dabieshanensis***sp. nov.** forms a phylogenetic lineage sister to the clade containing *C.parva* + *C.hypsibia.* The-Kimura 2-parameter genetic distances of the cytochrome b (CYT B) gene between the new species and other nominal *Chodsigoa* species ranged between 8.6 and 17.6%. The new species is distributed at elevations from 750 to 1250 m in the Dabie Mountains and is geographically distant from other species in the genus.

## ﻿Introduction

Asiatic shrews of the genus *Chodsigoa* Kastchenko, 1907 are mainly distributed in southwest China, adjacent Myanmar, Vietnam, and Thailand, and have also been recorded in central and eastern China and Taiwan (Hoffmann and Lunde 2008; Wilson and Mittermeier 2018). Animals in this genus are small in size (< 15 g) and mainly occur in mid-to high-montane forests, making them one of the least studied taxa among mammals. The genera *Chodsigoa* and Episoriculus were regarded as a subgenus of Soriculus (Hoffmann 1985) until recently, when Hutterer (2005) promoted them to full genus status. The most distinctive morphological characters distinguishing *Chodsigoa* from *Soriculus*/*Episoriculus* is the number of upper unicuspids. *Chodsigoa* has three upper unicuspids while *Soriculus*/*Episoriculus* has four. Nine species are currently recognized in *Chodsigoa*: *C.caovansunga* Lunde, Musser & Son, 2003, *C.furva* Anthony, 1941, *C.hoffmanni* Chen, He, Huang, Wan, Lin, Liu & Jiang, 2017, *C.hypsibia* (De Winton in De Winton and Styan 1899), *C.parca* Allen, 1923, *C.parva* Allen, 1923, *C.salenskii* (Kastschenko 1907), *C.smithii* Thomas, 1911 (Thomas 1911a), and *C.sodalis* Thomas, 1913.

The De Winton’s shrew (*C.hypsibia*) is endemic to China and is the most widely distributed species (Jiang and Hoffmann 2005). This gray, long-tailed shrew was first described by De Winton (1989) based on specimens from Yangliu-pa (= Yangliu ba), Pingwu, in Sichuan province. It contains two subspecies: *C.h.hypsibia*, recorded in Qinghai, Sichuan, Shaanxi, Tibet, Yunnan, Anhui, and Henan provinces (Zhang et al. 2018; Zhou et al. 2020) and *C.h.larvarum* Thomas, 1911 (Thomas 1911b), recorded in Beijing, Hebei, and Shanxi provinces (Liu et al. 2011). Zhang et al. (2018) reported the first record of *C.hypsibia* in Anhui province based on a specimen collected from Yaoluoping National Nature Reserve, Dabie Mountains. However, the collection site is distant from the known distribution of *C.hypsibia*, and the genetic distance of the CYT B gene between the specimen and *C.hypsibia* from Sichuan and Shaanxi (near the type locality in Pingwu, Sichuan) is relatively high (8.4–8.5%), and the two populations form deeply diverged clades in the Bayesian tree (posterior probabilities = 1.00; Zhang et al. 2018). These results suggest that additional studies with more specimens were necessary to confirm the taxonomic status of the population from the Dabie Mountains.

For three years, we conducted extensive field surveys in the Dabie Mountains, during which we collected 11 specimens of *Chodsigoa*. Based on morphological and molecular phylogenetic analysis, we recognize the population from the Dabie Mountains as distinct from *C.hypsibia* and other known *Chodsigoa* species, representing a new species *Chodsigoadabieshanensis* sp. nov., which we describe herein.

## ﻿Materials and methods

**Figure 1.**
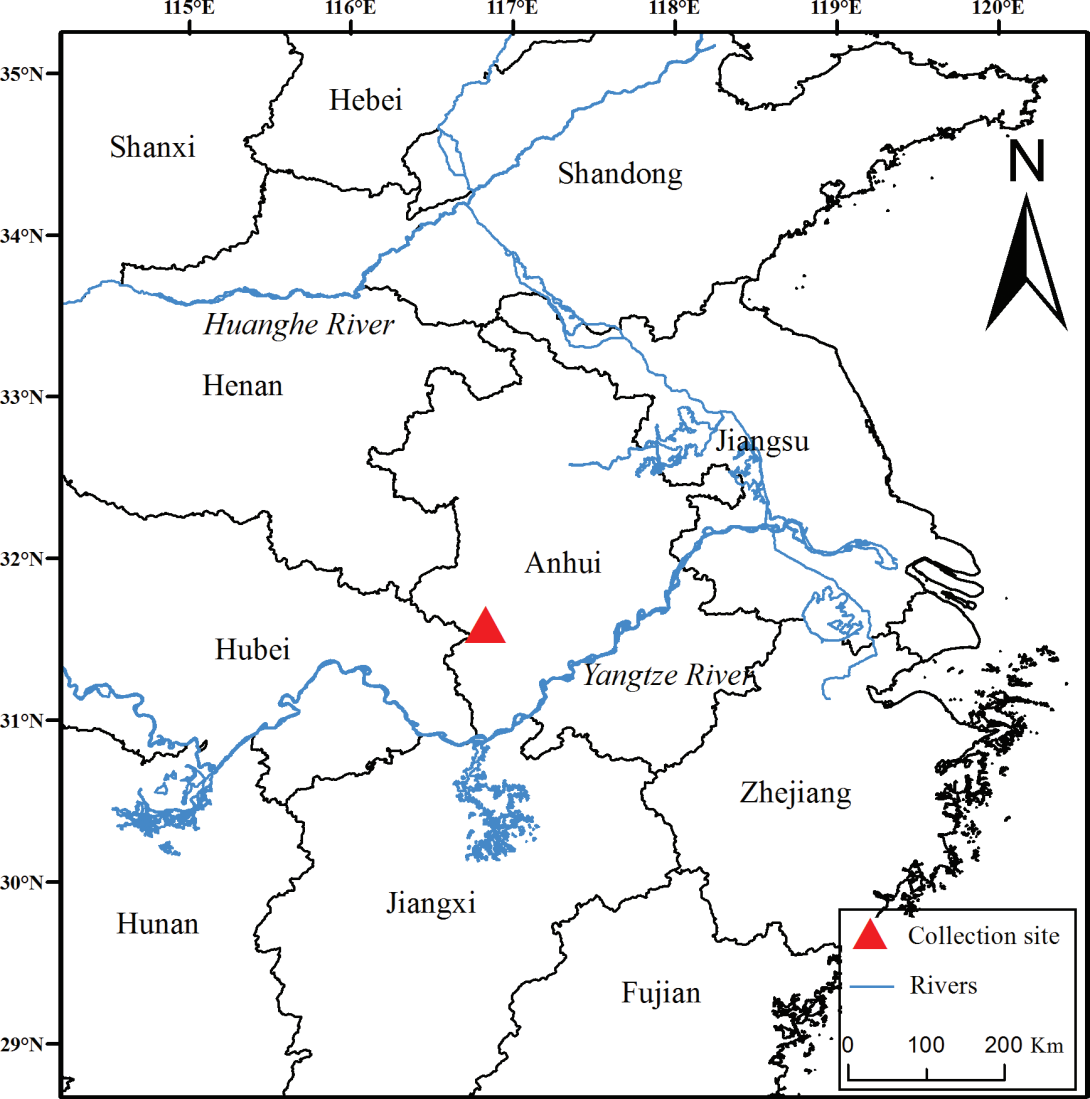
Map showing the collection site of *Chodsigoadabieshanensis* sp. nov. in the Dabie Mountains, Anhui Province, eastern China.

A total of 11 *Chodsigoa* specimens were collected from May 2017 to August 2020 from Yaoluoping National Nature Reserve (*n* = 1), Bancang Natural Reserve (*n* = 4), and Foziling Natural Reserve (*n* = 6), all located in the Dabie Mountains, Anhui province, eastern China (Fig. 1). Shrews were sampled using the pitfalls (plastic buckets 15 cm in diameter and 28 cm in depth). Specimens were euthanized and liver or muscle tissues were extracted and preserved in pure ethanol. Skulls were also extracted and cleaned. Specimens and tissues were deposited at the Biological Museum of Anhui University (**BMAHU**). Animals were handled consistent with the animal care and use guidelines of the American Society of Mammologists (Sikes et al. 2016), and also following the guidelines and regulations approved by the internal review board of Anhui Normal University, and with the permissions of local authorities.

External measurements including head and body length (**HB**), tail length (**TL**), hindfoot length (**HF**), ear length (**EL**) were taken in the field with a ruler to the nearest 0.1 mm. The body weight (**W**) of each specimen was weighed to the nearest 0.01 g using an electronic scale. All craniodental measurements were taken by CZZ using digital calipers graduated to the nearest 0.01 mm following Heaney and Timm (1983), Woodman and Timm (1993), and Chen et al. (2017). The following 19 measurements were taken:

**CIL** condyloincisive length;

**IOB** interorbital breadth;

**CB** cranial breadth;

**CH** cranial height;

**RL** rostral length;

**PRL** postrostral length;

**PIL** palatoincisive length;

**PPL** postpalatal length;

**UTL** upper toothrow length;

**M^2^–M^2^** maximum width across the upper second molars;

**P^4^–M^3^** distance from the upper fourth premolar to the upper third molar;

**PPD** postpalatal depth;

**BMF** foramen magnum breadth;

**ML** mandibular length;

**LTR** lower toothrow length;

**LLI** length of lower incisor;

**HCP** height of coronoid process;

**HCV** height of coronoid valley;

**HAC** height of articular condyle.

Comparative morphological data of another 149 *Chodsigoa* specimens were obtained from our previous study (Chen et al. 2017), including *C.caovansunga* (3), *C.furva* (5), *C.hoffmanni* (14), *C.hypsibia* (64), *C.parca* (19), *C.parva* (31), *C.smithii* (11), and *C.sodalis* (2).

To evaluate the morphological variation among populations of *Chodsigoa*, we performed a principal component analysis (**PCA**) in SPSS 19.0 (SPSS Inc., USA) using the log_10_-transformed craniodental measurements. We compared the morphology of the putative new species with other *Chodsigoa* species stored in Kunming Institute of Zoology (**KIZ**), the Sichuan Academy of Forestry (**SAF**), the Museum of Comparative Zoology, Harvard University (**MCZ**), and the American National Museum of Natural History (**AMNH**). The terminology for morphological descriptions followed Hoffman (1985), Lunde et al. (2003), and Chen et al. (2017).

Total genomic DNA of 10 *C.dabieshanensis* specimens were extracted using a DNA extraction kit (Qiagen DNeasy Blood and Tissue Kit, China). The complete CYT B gene and two nuclear gene segments [apolipoprotein B (APOB) and breast cancer 1 (BRCA1)] were amplified using primers and PCR conditions from Chen et al. (2021). The PCR products were purified and sequenced in both directions using the BigDye Terminator Cycle kit v. 3.1 (Invitrogen, USA) on an ABI 3730xl sequencer (Applied Biosystems, USA). Corresponding sequences of other *Chodsigoa* species were downloaded from GenBank (Table 1) and aligned with our new sequences using MUSCLE (Edgar 2004) and then checked manually by eye. Sequences of *Episoriculuscaudatus* (Horsfield, 1851) and *Neomysfodiens* (Pennant, 1771) were included in the alignments as outgroup taxa. The Kimura-2-parameter (K2P) distances of the CYT B gene between species were calculated in MEGA 7 (Kumar et al. 2016).

**Table 1.** Samples and sequences used for molecular analyses. New sequences generated in this study are shown in bold.

Species	Museum code	Collecting site	CYT B	BRCA1	APOB
*Chodsigoadabieshanensis*	AHUDBS017001	China: Anhui	MG462711	**OM200122**	**OM200113**
*Chodsigoadabieshanensis*	AHUDBS017002	China: Anhui	**OM200132**	**OM200123**	**OM200115**
*Chodsigoadabieshanensis*	AHUDBS017003	China: Anhui	**OM200131**	**OM200124**	**OM200114**
*Chodsigoadabieshanensis*	AHUDBS017004	China: Anhui	**OM200130**	**OM200125**	**OM200116**
*Chodsigoadabieshanensis*	AHU2008FZL001	China: Anhui	**OM200133**	**OM200121**	**OM200112**
*Chodsigoadabieshanensis*	AHU2008FZL002	China: Anhui	**OM200129**	**OM200120**	N.A.
*Chodsigoadabieshanensis*	AHU2008FZL003	China: Anhui	**OM200127**	**OM200119**	**OM200111**
*Chodsigoadabieshanensis*	AHU2008FZL004	China: Anhui	**OM200128**	N.A	**OM200110**
*Chodsigoadabieshanensis*	AHU2008FZL005	China: Anhui	**OM200126**	**OM200118**	**OM200109**
*Chodsigoadabieshanensis*	AHU2008FZL006	China: Anhui	N.A.	**OM200117**	**OM200108**
*Chodsigoacaovansunga*	KIZ:027112	China: Yunnan	JX508288	KX765593	KX765546
*Chodsigoacaovansunga*	AMNH:101500	Viet Nam: Ha Giang	AB175103	DQ630263	DQ630182
*Chodsigoacaovansunga*	AMNH:101520	Viet Nam: Ha Giang	AB175104	DQ630265	DQ630184
*Chodsigoafurva*	KIZ:032216	China: Yunnan	KX765525	KX765617	KX765571
*Chodsigoafurva*	KIZ:032217	China: Yunnan	KX765526	KX765618	KX765572
*Chodsigoahypsibia*	KIZ:021075	China: Yunnan	KX765534	KX765625	KX765581
*Chodsigoahypsibia*	KIZ:021483	China: Yunnan	KX765536	KX765626	KX765583
*Chodsigoahypsibia*	KIZ:021485	China: Yunnan	KX765535	KX765627	KX765582
*Chodsigoahypsibia*	KIZ:032302	China: Sichuan	KX765527	KX765637	KX765575
*Chodsigoahypsibia*	KIZ:032250	China: Qinghai	KX765528	KX765624	KX765574
*Chodsigoahypsibia*	KIZ:032251	China: Qinghai	KX765529	KX765630	KX765577
*Chodsigoaparca*	KIZ:032246	China: Yunnan	KX765502	KX765600	KX765551
*Chodsigoaparca*	KIZ:032239	China: Yunnan	KX765504	KX765607	KX765549
*Chodsigoaparca*	KIZ:032243	China: Yunnan	GU981265	KX765602	KX765550
*Chodsigoaparva*	KIZ:032235	China: Yunnan	KX765539	KX765631	KX765586
*Chodsigoaparva*	KIZ:022222	China: Yunnan	KX765542	KX765632	KX765591
*Chodsigoaparva*	KIZ:020265	China: Yunnan	KX765543	KX765633	KX765589
*Chodsigoasmithii*	SAF: BLG012	China: Sichuan	KX765521	KX765609	KX765567
*Chodsigoasmithii*	SAF: BLG144	China: Sichuan	KX765522	KX765610	KX765568
*Chodsigoasmithii*	SAF: JJSA616	China: Sichuan	KX765524	KX765612	KX765562
*Chodsigoasodalis*	JUM016	China: Taiwan	AB175102	DQ630274	DQ630194
*Chodsigoasodalis*	T0497	China: Taiwan	AB127978	DQ630271	DQ630191
*Chodsigoasodalis*	THUB-S-00007	China: Taiwan	GU981270	GU981191	GU981116
*Chodsigoahoffmanni*	KIZ:019442	China: Yunnan	KX765509	KX765594	KX765555
*Chodsigoahoffmanni*	KIZ:019458	China: Yunnan	KX765510	KX765595	KX765558
*Chodsigoahoffmanni*	KIZ:019459	China: Yunnan	KX765512	KX765596	KX765559
*Episoriculuscaudatus*	19716	China: Yunnan	GU981272	GU981193	GU981118
*Neomysfodiens*	65298	Germany	GU981295	GU981205	GU981130

Three datasets were used for the phylogenetic analyses: CYT B gene, concatenated nuclear genes, and concatenated mitochondrial and nuclear genes (Table 1). Maximum likelihood (**ML**) and Bayesian inference (**BI**) analyses were performed to reconstruct the phylogenetic relationships in PhyloSuite (Zhang et al. 2020) based on the best-fit partitioning schemes estimated using PartitionFinder v. 2.0 (Lanfear et al. 2012). The ultrafast bootstrap values (UFBoot) ≥ 95 and posterior probabilities (PP) ≥ 0.95 were considered as strong supports (Huelsenbeck and Rannala 2004; Minh et al. 2018).

## ﻿Results

**Figure 2.**
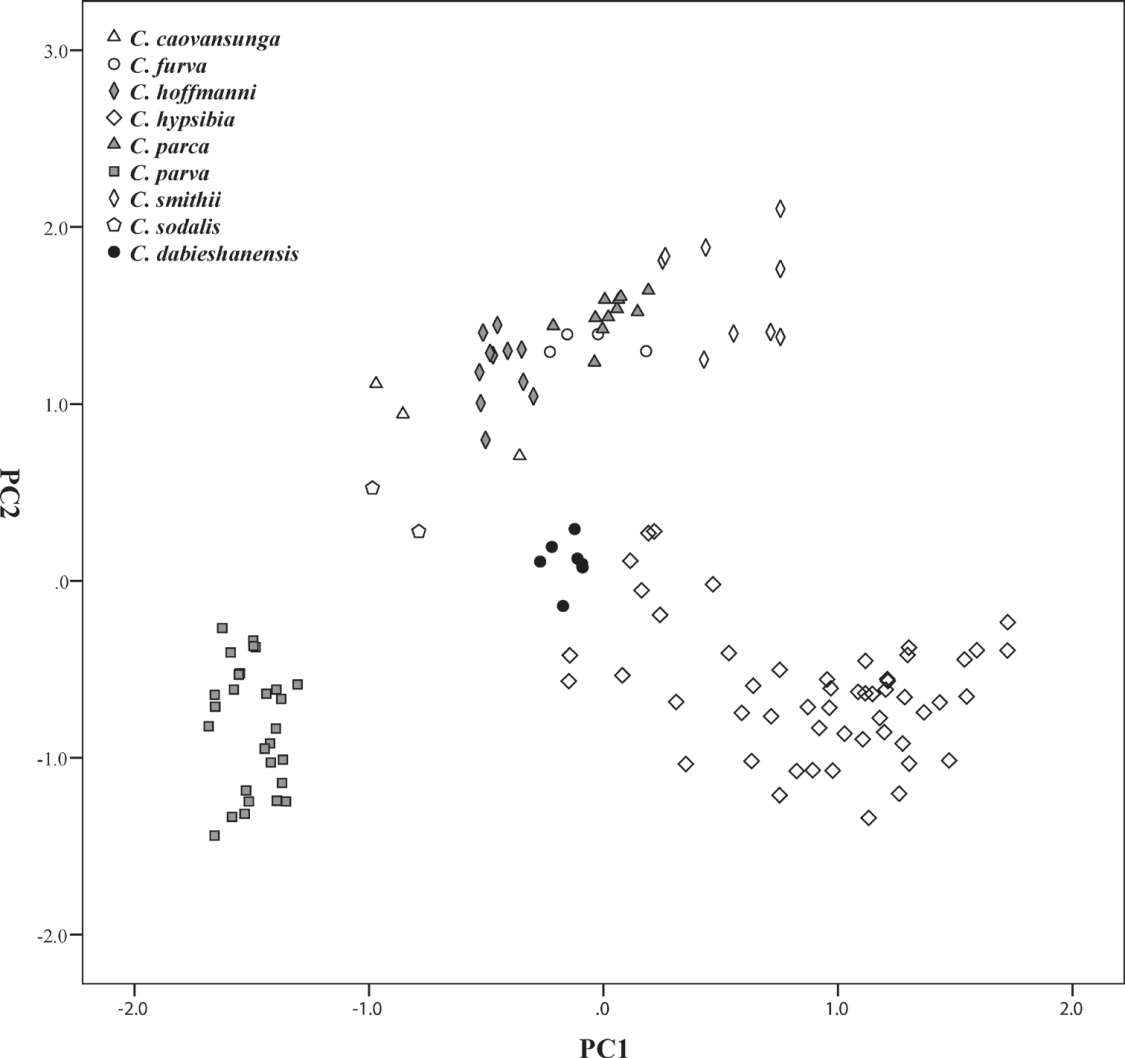
Results of principal component analysis of *Chodsigoa* based on the 19 log_10_-transformed craniodental measurements.

External and cranial measurements are summarized in Table 2. The PCA based on 128 intact skulls produced two axes with eigenvalues exceeding 1.0, which explained 94.2% of the variation (Table 3). The first axes (PC1) explained 86.2% of the variation and was strongly positively correlated with all variables, indicating it represented the overall skull size (Table 3). The second axis (PC2) explained 8.0% of the variation and was highly positively correlated with CH and BMF (loading > 0.67). A plot of PC1 and PC2 (Fig. 2) showing that *C.dabieshanensis* are separated well from all named species. This new species occurs in the center of the morphospace, indicating its medium size in the genus. Morphologically, it is most similar to *C.hypsibia*, with which it occupies the upper left corner morphospace without overlap (Fig. 2), indicating its generally smaller size, larger BMF, and higher CH (Table 2).

**Table 2.** External and craniomandibular measurements (mm), including mean values, standard deviations, ranges, and sample sizes of *Chodsigoa* species. The measurements were obtained from Chen et al. (2017), except for *C.dabieshanensis* sp. nov.

Variable	*C.dabieshanensis* sp. nov.	*C.caovansunga*	*C.furva*	*C.hypsibia*	*C.parca*	*C.hoffmanni*	*C.parva*	*C.smithii*	*C.sodalis*
	*N = 11*	*N* = 3	*N* = 5	*N* = 58	*N* = 16	*N* = 14	*N* = 31	*N* = 11	*N* = 2
W	5.24±0.36 4.67–5.89; 9	6.20; 1	6.05±0.64 5.60–6.50; 2	10.40±1.61 6.40–14.00; 30	9.35±1.09 7.90–11.90; 13	7.54±0.80 7.00–9.60; 12	3.59±0.56 2.60–5.20; 29	9.69±1.46 7.00–12.00; 10	
HB	67.22±3.23 62.00–73.00; 9	74.00; 1	71.67±3.06 69.00–75.00; 3	75.48±5.75 62.00–86.00; 52	70.30±4.40 62.00–77.00; 14	66.75±5.15 58.00–75.00; 12	56.66±4.33 47.00–64.00; 29	79.70±2.71 76.00–84.00; 10	55.50±2.12 54.00–57.00; 2
TL	59.67±3.28 54.00–64.00; 9	83.00; 1	86.00±1.73 84.00–87.00; 3	65.69±4.01 56.00–73.00; 52	90.60±5.70 77.00–99.00; 14	81.67±4.21 74.00–88.00; 12	44.90±8.23 4.60–52.00; 29	98.90±5.28 93.00–110.00; 10	57.50±3.54 55.00–60.00; 2
HF	13.44±0.53 13.00–14.00; 9	15.00; 1	17.33±1.15 16.00–18.00; 3	15.35±1.17 13.00–18.00; 53	16.50±0.90 15.00–18.00; 15	15.50±0.80 14.00–17.00; 12	10.81±0.51 10.00–12.00; 29	17.90±1.13 16.00–20.00; 10	13.00±0.00 13.00–13.00; 2
EL	8.22±0.44 8.00–9.00; 9	9.00; 1	8.00±0.00 8.00–8.00; 2	7.04±1.12 5.00–9.50; 37	8.89±1.24 7.00–11.50; 14	8.83±1.11 7.00–11.00; 12	6.93±0.54 5.00–8.00; 28	8.89±1.96 6.00–12.00; 9	8.50±0.71 8.00–9.00; 2
CIL	19.08±0.22 18.65–19.26; 8	17.96±0.74 17.38–18.80; 3	20.63±0.39 20.16–21.06; 4	20.66±0.89 19.03–22.62; 46	20.37±0.29 20.08–20.88; 8	19.13±0.39 18.31–19.57; 12	15.79±0.27 15.08–16.17; 29	22.23±0.54 21.50–23.05; 9	17.97±0.12 17.88–18.05; 2
IOB	4.52±0.07 4.41–4.62; 8	4.30±0.06 4.23–4.35; 3	4.96±0.10 4.85–5.05; 4	5.04±0.33 3.99–5.56; 51	4.77±0.11 4.60–4.99; 10	4.40±0.13 4.14–4.58; 12	3.55±0.15 3.25–3.85; 29	5.23±0.21 4.86–5.48; 9	4.10±0.15 3.99–4.20; 2
CB	9.01±0.18 8.81–9.37; 9	8.78±0.08 8.71–8.87; 3	9.38±0.34 9.10–9.84; 4	9.42±0.40 8.38–10.34; 49	9.57±0.14 9.33–9.82; 10	9.06±0.25 8.45–9.39; 12	7.30±0.22 6.93–7.73; 29	9.95±0.25 9.67–10.45; 9	8.14±0.45 7.82–8.46; 2
CH	4.96±0.18 4.67–5.23; 9	5.24±0.28 5.05–5.57; 3	5.67±0.29 5.45–6.09; 4	4.57±0.28 4.05–5.10; 47	5.95±0.15 5.71–6.19; 10	5.61±0.16 5.30–5.87; 12	4.02±0.19 3.71–4.32; 29	6.09±0.16 5.87–6.30; 9	4.74±0.14 4.64–4.84; 2
RL	6.61±0.11 6.48–6.81; 8	6.43±0.58 6.04–7.10; 3	7.76±0.17 7.57–7.91; 4	7.72±0.46 6.93–9.00; 52	7.83±0.15 7.55–7.98; 9	7.29±0.19 6.78–7.56; 12	5.63±0.16 5.33–6.07; 29	8.78±0.35 8.14–9.18; 9	6.70±0.01 6.69–6.70; 2
PRL	11.84±0.18 11.56–12.04; 8	10.86±0.67 10.09–11.27; 3	12.35±0.48 11.93–12.84; 4	12.97±0.61 11.55–14.23; 46	12.24±0.18 12.06–12.55; 9	11.57±0.28 11.02–11.96; 12	9.87±0.18 9.32–10.14; 29	13.29±0.29 12.93–13.80; 9	10.79±0.15 10.68–10.89; 2
PIL	8.36±0.16 8.08–8.49; 8	7.96±0.30 7.76–8.31; 3	8.97±0.24 8.76–9.30; 4	9.17±0.51 8.05–10.37; 52	9.08±0.14 8.90–9.28; 9	8.43±0.18 8.06–8.75; 12	6.61±0.13 6.38–6.85; 29	9.92±0.37 9.40–10.50; 9	7.95±0.06 7.91–7.99; 2
PPL	8.85±0.12 8.63–8.97; 8	8.11±0.43 7.80–8.60; 3	9.28±0.34 8.89–9.59; 4	9.55±0.41 8.87–10.78; 46	9.11±0.19 8.77–9.35; 10	8.79±0.18 8.57–9.11; 12	7.60±0.19 7.10–7.90; 29	10.03±0.36 9.67–10.84; 9	8.15±0.01 8.14–8.15; 2
UTL	8.05±0.11 7.85–8.19; 8	7.76±0.25 7.58–8.05; 3	8.86±0.25 8.57–9.18; 4	8.50±0.38 7.88–9.42; 52	8.85±0.12 8.59–9.02; 9	8.11±0.16 7.68–8.31; 12	6.44±0.14 6.11–6.67; 29	9.70±0.38 9.01–10.20; 9	7.73±0.06 7.69–7.77; 2
M^2^–M^2^	5.56±0.09 5.42–5.66; 8	5.13±0.11 5.06–5.26; 3	5.58±0.16 5.39–5.75; 4	6.04±0.34 5.34–6.74; 52	5.36±0.09 5.26–5.51; 10	5.22±0.08 5.12–5.36; 12	4.24±0.19 3.92–4.53; 29	5.92±0.15 5.75–6.24; 9	4.49±0.18 4.36–4.62; 2
P^4^–M^3^	4.89±0.05 4.82–4.95; 8	4.65±0.10 4.57–4.77; 3	5.39±0.22 5.07–5.56; 4	5.27±0.26 4.66–5.86; 52	5.71±0.09 5.57–5.84; 10	4.82±0.11 4.59–5.03; 12	3.94±0.12 3.57–4.12; 29	5.78±0.24 5.47–6.10; 9	4.85±0.04 4.82–4.88; 2
PPD	2.81±0.10 2.64–2.95; 8	3.25±0.08 3.18–3.34; 3	3.50±0.09 3.40–3.59; 4	3.07±0.19 2.66–3.37; 51	3.90±0.09 3.72–3.98; 10	3.50±0.14 3.11–3.65; 12	2.47±0.13 2.20–2.69; 29	3.84±0.21 3.50–4.12; 9	3.05±0.08 2.99–3.11; 2
BMF	3.20±0.11 3.07–3.43; 9	3.17±0.07 3.11–3.24; 3	3.57±0.13 3.38–3.65; 4	2.76±0.14 2.53–3.21; 51	3.32±0.13 3.18–3.55; 9	3.26±0.09 3.12–3.44; 12	2.57±0.17 2.22–2.86; 29	3.71±0.24 3.40–4.20; 9	2.99±0.01 2.98–2.99; 2
ML	10.05±0.17 9.74–10.29; 9	10.06±0.33 9.79–10.43; 3	11.07±0.29 10.79–11.35; 4	10.94±0.51 10.18–12.37; 54	11.45±0.17 11.13–11.72; 10	10.60±0.19 10.31–10.96; 12	8.33±0.18 7.97–8.76; 28	12.20±0.42 11.70–12.90; 9	9.66±0.32 9.43–9.88; 2
LTR	7.41±0.25 7.21–8.09; 9	7.25±0.14 7.12–7.39; 3	8.06±0.20 7.88–8.26; 4	8.10±0.42 7.31–9.12; 53	8.15±0.13 7.96–8.34; 10	7.50±0.14 7.19–7.67; 12	5.95±0.13 5.70–6.23; 28	8.78±0.34 8.30–9.20; 9	6.95±0.35 6.70–7.20; 2
LLI	3.27±0.06 3.22–3.42; 9	3.19±0.15 3.06–3.36; 3	3.17±0.20 2.89–3.35; 4	3.67±0.30 2.70–4.25; 53	3.42±0.16 3.07–3.62; 10	3.23±0.09 3.08–3.37; 12	2.53±0.15 2.25–2.78; 28	3.65±0.19 3.25–3.90; 9	2.71±0.21 2.56–2.86; 2
HCP	3.94±0.12 3.71–4.09; 9	4.00±0.06 3.93–4.05; 3	3.98±0.12 3.88–4.12; 4	4.35±0.30 3.85–5.09; 54	4.64±0.11 4.52–4.81; 10	4.06±0.15 3.70–4.36; 12	2.96±0.17 2.63–3.31; 28	4.37±0.29 3.90–4.72; 9	3.43±0.03 3.41–3.45; 2
HCV	2.34±0.08 2.21–2.46; 9	2.61±0.01 2.60–2.62; 3	2.65±0.09 2.56–2.77; 4	2.71±0.26 2.20–3.32; 54	3.01±0.10 2.87–3.26; 10	2.66±0.07 2.56–2.80; 12	1.96±0.10 1.77–2.19; 28	2.95±0.15 2.80–3.20; 9	2.33±0.01 2.32–2.33; 2
HAC	2.85±0.10 2.70–2.98; 9	3.31±0.02 3.30–3.34; 3	3.45±0.11 3.31–3.57; 4	3.43±0.27 2.87–4.02; 46	3.67±0.06 3.59–3.79; 10	3.45±0.13 3.24–3.66; 12	2.48±0.12 2.18–2.68; 28	3.78±0.15 3.60–4.00; 9	2.92±0.10 2.85–2.99; 2

**Table 3.** Character loadings, eigenvalues, and proportion of variance explained by the first two axes (PC1 and PC2) of a principal component analysis using the log_10_-transformed measurements of *Chodsigoa*. The meanings of variable abbreviations are given in the Materials and methods section.

Variables	Principal component	
	1	2
ML	0.991	0.047
PIL	0.990	–0.085
LTR	0.988	–-0.073
CIL	0.987	–0.107
UTL	0.986	0.060
P^4^–M^3^	0.982	–0.057
CB	0.977	–0.009
RL	0.972	–0.030
HCP	0.961	–0.052
IOB	0.955	–0.200
PRL	0.949	–0.262
HCV	0.940	0.078
HAC	0.939	0.075
PPL	0.937	–0.221
M^2^–M^2^	0.932	–0.259
LLI	0.910	–0.269
PPD	0.841	0.464
CH	0.692	0.670
BMF	0.610	0.713
Eigenvalue	16.385	1.519
Variance explained	86.235	7.993

**Figure 3.**
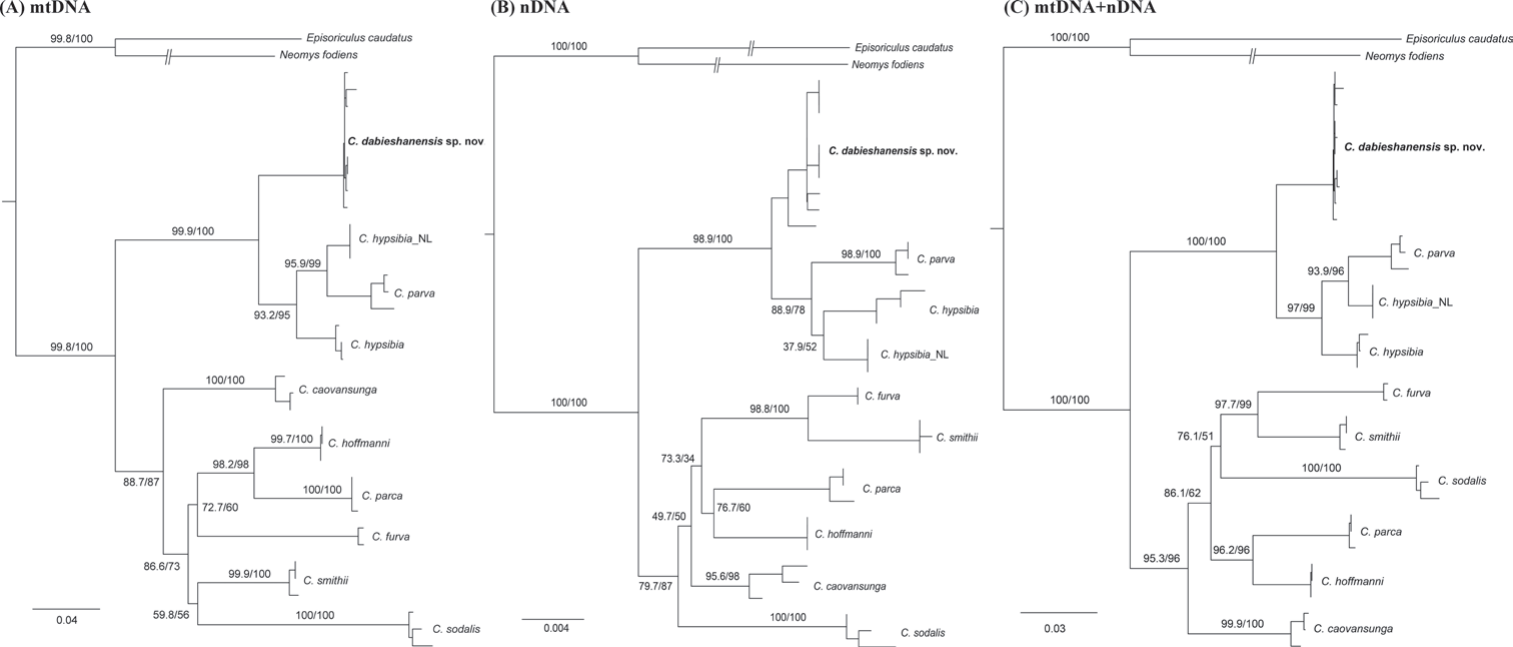
Maximum likelihood phylogenetic trees derived from **A** the CYT B gene **B** the concatenated nuclear genes **C** the concatenated mitochondrial-nuclear trees. Branch labels indicate Bayesian posterior probabilities (PP) and ultrafast bootstrap supports (UFBoot). Scale bars represent substitutions per site.

Nine CYT B (1140 bp), nine APOB (513 bp), and nine BRCA1 (768 bp) sequences of *C.dabieshanensis* were obtained (GenBank accession numbers: OM200108–OM200133; Table 1). The ML and BI trees recovered very similar topologies, and therefore, only the ML gene trees are shown (Fig. 3). The phylogenetic analyses of all three datasets supported *Chodsigoa* clustered into two major clades (UFboot > 99, PP = 1.00). One clade was composed of *C.parva*, *C.hypsibia*, and *C.dabieshanensis* (Clade I), and the other clade was composed of *C.caovansunga*, *C.furva*, *C.hoffmanni*, *C.parca*, *C.salenskii*, *C.smithii*, and *C.sodalis* (Clade II). The *C.dabieshanensis* clade was strongly supported as a monophyletic lineage, sister to the clade containing *C.parva* and *C.hypsibia* (UFboot > 98, PP = 1.00). The K2P genetic distances of the CYT B gene between *C.dabieshanensis* and other nominal *Chodsigoa* species ranged from 8.6% (with *C.hypsibia*) to 17.6% (with *C.sodalis*) (Table 4).

**Table 4.** The Kimura-2-parameter distances between *Chodsigoa* species based on the CYT B gene.

	*C.dabieshanensis* sp. nov.	*C.caovansunga*	*C.furva*	*C.hoffmanni*	*C.hypsibia*	*C.parca*	*C.parva*	*C.smithii*
*C.dabieshanensis* sp. nov.	–	–	–	–	–	–	–	–
*C.caovansunga*	0.147	–	–	–	–	–	–	–
*C.furva*	0.151	0.131	–	–	–	–	–	–
*C.hoffmanni*	0.147	0.116	0.132	–	–	–	–	–
*C.hypsibia*	0.086	0.144	0.155	0.146	–	–	–	–
*C.parca*	0.152	0.128	0.131	0.082	0.152	–	–	–
*C.parva*	0.102	0.154	0.162	0.154	0.058	0.160	–	–
*C.smithii*	0.163	0.112	0.119	0.104	0.153	0.122	0.164	–
*C.sodalis*	0.176	0.144	0.155	0.136	0.162	0.140	0.162	0.131

Based on the morphological, morphometric, and molecular evidence and the modern phylogenetic species concept (phylogenetic species concept based on both diagnosability and monophyly as operational criteria) (Mayden 1997; Gutierrez and Garbino 2018), we recognize the population from the Dabie Mountains as a new species of *Chodsigoa*, which is formally described below.

## ﻿Taxonomic account

### ﻿ Chodsigoa dabieshanensis sp. nov.

Taxon classificationAnimaliaEulipotyphlaSoricidae

AFACD42E-DC6C-5657-8612-A6C37DF62B8B

http://zoobank.org/A2EF195A-A19C-43CD-A774-A06218E96EE9

**Figure 4.**
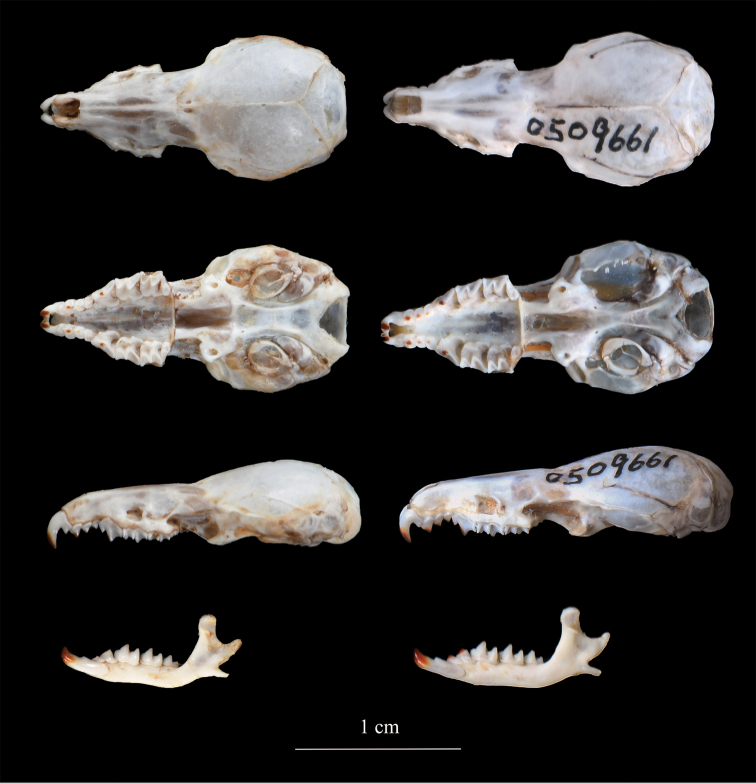
Dorsal, ventral, and lateral views of the skull and lateral views of the mandible of the holotype of *Chodsigoadabieshanensis* sp. nov. (AHU2008FZL004; left) and *Chodsigoahypsibia* (KIZ 016077; right). Scale bar: 10 mm.

**Figure 5.**
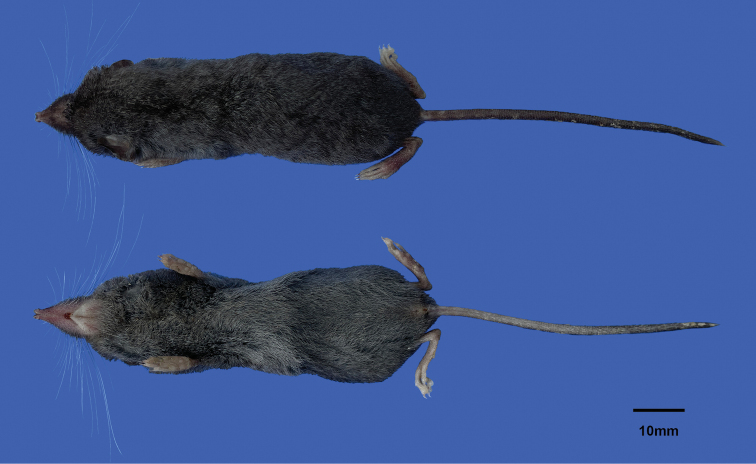
Dorsal and ventral view of *Chodsigoadabieshanensis* sp. nov.

Figures 4 , 5 , Table 2

#### Suggested common name.

Dabieshan long-tailed shrew; 大别山缺齿鼩 (Dabieshan Quechiqu)

***Holotype.*** AHU2008FZL005, an adult female collected by Zhen Xu and Ruolei Sun in August 2020, at Foziling natural reserve (31°07'07"N, 116°14'41"E, 1187 m a.s.l.), the north slope of the Dabie Mountains, Huoshan County, Luan City, Anhui province, China. Cleaned skulls and remaining carcasses frozen at –20 °C deposited in the Biological Museum of Anhui University (BMAHU).

***Paratypes.*** AHUDBS017001-005; AHU2008FZL001-004, 006. Ten specimens collected between May 2017 and August 2020 from the Dabie Mountains, Anhui province, China. All specimens are deposited in the Biological Museum of Anhui University (BMAHU).

#### Etymology.

The specific name *dabieshanensis* is derived from the Dabie Mountains, the type locality of the new species: -*shan* means mountain in Chinese, and the Latin adjectival suffix -*ensis* means “belonging to".

#### Diagnosis.

The new species is assigned to the genus *Chodsigoa* for having three upper unicuspid teeth, with the tips of the teeth lightly pigmented (Fig. 4). *Chodsigoadabieshanensis* sp. nov. can be distinguished from the other known species of *Chodsigoa* by the following combination of characters: small to medium in size (HB = 67.22 mm; CIL = 19.08 mm), dark brownish pelage; tail shorter than the HB, nearly similar ventral and dorsal pelage color, a small tuft of longer hairs at the tip of the tail (Fig. 5); markedly flattened braincase; and the foramen magnum is relatively wider than *C.hypsibia*. Phylogenetic analyses show that the new species is monotypic, sister to *C.hypsibia* and *C.parva* (Fig. 3).

#### Description.

A small to medium-sized shrew (W = 5.24±0.36 g, range 4.67–5.89 g; HB = 67.22±3.23 mm, range 62.00–73.00 mm, Table 2) with dark brown dorsal pelage and slightly paler ventral pelage (Fig. 5). Tail is short (TL = 59.67±3.28 mm), about 90% of the head and body length, brown above, slightly paler below, and with a small tuft of longer hairs at the tip. External ears are prominent, rounded, and covered with very short dark hairs. Eyes are very small. The dorsal surfaces of hands and hind feet are covered with short brown hair, lighter at the margin. The thenar and hypothenar pads at the soles of the hindfeet are well separated.

The skull of *C.dabieshanensis* sp. nov. is short and broad, and the braincase is markedly flattened (Fig. 4). The skull is similar to *C.hypsibia*, but much shorter and broader. The rostrum is short, and the interorbital region is wide. From the ventral view, the rostrum gradually narrows in the premaxillary region. The palate is short, with an abrupt posterior edge. The basisoccipital is developed and the ridges are approximately parallel. The dentition is the same for the genus: 3.1.1.3/2.0.1.3 = 28. The first incisor is long, falciform; the apex straight downwards; the talon much lower than U^1^, approximately equal to U^3^. Three upper unicuspids are present. All unicuspids are crowded and overlap slightly at the base. U^1^–U^3^ gradually decrease in size; U^3^ is about half as high as U^1^, and in contact with P^4^, which is large and triangular in outline. The posterior borders of P^4^ and M^1^ are deeply excavated, appearing crescent, while the posterior borders of M^2^ are much shallower. M^3^ is reduced and much narrower with a single lobe. The tips of the anterior teeth have a lightly pigmented chestnut color except the molars.

The mandible is slender. The coronoid process is tall and squared, rising straight upward from the posterior of the toothrow. The condyloid process is weak and bi-faceted, forming an angle at approximately 45° with the coronoid process. The angular process is long, straight, and very thin. The first lower incisor is long, with only a single basal cusplet. The incisor is slightly curved upwards, forming a hook at the tip. The first lower unicuspid is small and procumbent, crowded with a large incisor and the following premolar. The premolar has one forward-leaning cusp. The molar gradually decreases in size from M^1^ to M^3^. Only the tips of I_1_, U_1_, P_1_, and M_1_ are chestnut-pigmented but not those of M_2_ and M_3._

#### Comparison.

Among the species in the genus *Chodsigoa*, *C.dabieshanensis* sp. nov. is morphologically similar to the widely distributed *C.hypsibia*. However, the new species can be distinguished from *C.hypsibia* by many characters. In terms of body size, *C.dabieshanensis* sp. nov. is much smaller than *C.hypsibia* for most external and craniomandibular measurements (Table 2). In particular, the range of weight (4.67–5.89 g vs 6.40–14.00 g) and rostral length (6.48–6.81 mm vs 6.93–9.00 mm) between the two species does not overlap. The overall pelage of *C.dabieshanensis* sp. nov. is dark brown, almost black, which differs from the gray pelage of *C.hypsibia.* The skull of *C.dabieshanensis* sp. nov. is relatively shorter and broader than *C.hypsibia*, especially in the interorbital region, which appears much flatter (Fig. 4). The foramen magnum breadth is relatively larger than *C.hypsibia.* The posterior borders of M_2_ in *C.hypsibia* are much more deeply excavated than in *C.dabieshanensis* sp. nov.. In *C.dabieshanensis* sp. nov., the basioccipital is well developed and the ridges are approximately parallel. By contrast, the basioccipital of *C.hypsibia* is narrow, so the ridges are nearly confluent in the middle.

*Chodsigoadabieshanensis* sp. nov. (CIL = 19.08±0.22 mm) can be easily distinguished from *C.parva* (CIL = 15.79±0.27 mm) by its much larger size and the ranges of most of their external and cranial measurements do not overlap (Table 2). Furthermore, the tail of *C.dabieshanensis* sp. nov. (TL/HB = 80%) is relatively longer than *C.parva* (TL/HB = 88%). If the mean condyloincisive length is used as an indicator of overall size, *C.dabieshanensis* sp. nov. (CIL = 19.08±0.22 mm) is larger than *C.sodalis* (CIL = 17.97±0.12 mm), but smaller than *C.furva* (CIL = 20.63±0.39 mm), *C.parca* (CIL = 20.37±0.29 mm), and *C.smithii* (CIL = 22.23±0.54 mm) (Table 2). The markedly flattened cranium of *C.dabieshanensis* sp. nov. is clearly distinguished from all other species in the genus, including *C.caovansunga*, *C.furva*, *C.hoffmanni*, *C.parca*, *C.salenskii*, *C.smithii*, and *C.sodalis.* The tail of *C.dabieshanensis* sp. nov. is shorter than head and body length, and it differs from *C.sodalis* (TL/HB ≈ 100%) and all other *Chodsigoa* species (TL/HB > 100%). The new species has a tuft of longer hair at the tip of the tail, in contrast to *C.caovansunga*, *C.furva*, and *C.smithii*. The thenar and hypothenar pads at the soles of the hindfeet are well separated and distinguishable from *C.caovansunga*, whose thenar and hypothenar pads of hindfeet are close together.

#### Distribution and habits.

*Chodsigoadabieshanensis* sp. nov. is currently known from Yaoleping National Nature Reserve, Bancang Natural Reserve, and Foziling Natural Reserve, all located in the Dabie Mountains, Anhui province, eastern China. Most specimens were collected from deciduous broad-leaf forests at 750–1250 m a.s.l.

## ﻿Discussion

Prior to this study, nine species were recognized in the genus *Chodsigoa* (Chen et al. 2017; Wilson and Mittermeier 2018). Our morphological and molecular results support that the specimens from the Dabie Mountains represent a new species of *Chodsigoa*, C. *dabieshanensis* sp. nov., based on the diagnosis-and-monophyly-based phylogenetic species concept (Mayden 1997; Gutierrez and Garbino 2018). *Chodsigoadabieshanensis* sp. nov. is morphologically closely related to *C.hypsibia* and was previously considered as a marginal population of that taxon (Zhang et al. 2018). However, it can be distinguished from *C.hypsibia* by its dark brownish pelage and smaller size (Table 2). The large genetic distance (8.6% by the CYT B gene) and phylogenetic analysis also strongly support they are two distinct species (UFboot > 98, PP = 1.00). As *Chodsigoa* are mainly distributed in southwest China and adjacent areas (Wilson and Mittermeier 2018), the distribution area of *C.dabieshanensis* sp. nov. is marginal. It is the only known species of *Chodsigoa* recorded in Anhui province, separated by at least 500 km from any other member of the genus, i.e., C. *hypsibia* from Luanxian, Henan Province (Zhou et al. 2020). The new species has no known congeners in Anhui Province; there are only two other soricid taxa recorded, *Chimarrogalelender* Tomas, 1902 and *Crocidura* spp. (Wang 1990; Jiang et al. 2015). The former is a large aquatic shrew (W > 20 g), and the latter has white, unpigmented dentition; these taxa are easily distinguishable from the new species.

The new species brings the number of *Chodsigoa* species to 10, sorted into two major clades; one including *C.parva* + *C.hypsibia* + *C.dabieshanensis* sp. nov. (Clade I), and the other (Clade II) comprised of the remaining species (Fig. 3). These results are also supported by morphology. Compared with the species in Clade II, the cranium of Clade I species is markedly flatter, and the tail of Clade I is relatively shorter (Clade I: TL/HB < 100%; Clade II: TL/HB ≥ 100%). All our gene trees showed *C.dabieshanensis* sp. nov. forms a subclade inside the main Clade I as the sister group of the subclade *C.parva* + *C.hypsibia* (UFboot > 98, PP = 1.00, Fig. 3).

As the most easterly distributed species of *Chodsigoa*, the discovery of *C.dabieshanensis* sp. nov. from the Dabie Mountains is important in understanding the macroevolution of the genus. Previous studies suggested that the tribe Nectogalini originated from Europe and migrated eastward to western Siberia and southward along northern China to southwest China (He et al. 2010). While the Hengduan Mountains are considered to serve as an important route for the southward migration (Zhang 2002; He et al. 2010), we have no knowledge of how this group migrated eastward. The oldest fossils of *Chodsigoa* are from the Early Pliocene in Gansu Provence, northern China (Zhang and Zheng 2001). Fossils of C.cf.hypsibia and C.cf.parva were discovered from the Early Pleistocene in Jianshi, Hubei and Wuhu, Anhui, both in eastern China, and more fossils were found in Wushan, Chongqing, southwest China in the Late Pleistocene (Qiu and Li 2005). These fossil records, together with our finding of *C.dabieshanensis* sp. nov., diverged earlier than *C.hypsibia* and *C.parva*, which suggests that the ancestor of Clade I arrived early in eastern China. Due to the present lack of broad geographic sampling, how the genus migrated to eastern China is still an open question. The Dabie Mountains are an extension of the Qinling fold belt and gradually stabilized by the end of the Tertiary (Feng 1976). Considering that the montane archipelagos always act as refugia and corridors to facilitate the dispersal of terrestrial small mammals (Chen et al. 2015; He and Jiang 2014; He et al. 2019), a parsimonious biogeographic scenario of the migration is via the Qinling and Dabie mountains. The ancestor of new species then became isolated due to climate change and following habitat turnover, resulting in a new species. Finer taxon sampling with additional sequence data is warranted to illustrate the migration patterns of the genus.

## Supplementary Material

XML Treatment for Chodsigoa dabieshanensis
